# Comparison of Corresponding Scores From the Cleft Hearing Appearance and Speech Questionnaire (CHASQ) and CLEFT-Q in Swedish Patients With Cleft Lip and/or Palate

**DOI:** 10.1177/1055665620964124

**Published:** 2020-10-13

**Authors:** Mia Stiernman, Kristina Klintö, Martin Persson, Magnus Becker

**Affiliations:** 1 Department of Plastic and Reconstructive Surgery, Skåne University Hospital, Malmö, Sweden; 2 Department of Clinical Sciences in Malmö, 5193 Lund University, Sweden; 3 Department of Otorhinolaryngology, Skåne University Hospital, Malmö, Sweden; 4 Department of Health and Society, Kristianstad University, Sweden

**Keywords:** patient-reported outcomes, cleft lip and/or cleft palate, CHASQ, CLEFT-Q

## Abstract

**Objective:**

The primary aim of this study was to compare corresponding scores between 2 existing cleft-specific patient-reported outcome measures (PROMs)—Cleft Hearing Appearance and Speech Questionnaire (CHASQ) and CLEFT-Q. The second aim of the study was to investigate patient opinion on the 2 PROMs.

**Design:**

Cross-sectional questionnaire study.

**Setting:**

Participants were recruited from a University Hospital. They answered CHASQ and CLEFT-Q either in the hospital or at home.

**Participants:**

Thirty-three participants with cleft lip and/or palate, aged 10 to 19 years.

**Main Outcome Measure:**

CHASQ and CLEFT-Q.

**Results:**

The CHASQ scores and the corresponding CLEFT-Q scores on appearance correlated significantly. Corresponding scores regarding speech did not correlate significantly. A majority, 15 (58%) participants, answered that they liked CLEFT-Q more than CHASQ, 18 participants (69%) thought CHASQ was easier to complete, and 19 (76%) thought CLEFT-Q would better inform health care professionals.

**Conclusion:**

Both instruments showed strengths and limitations. Clinicians will have to consider each instrument’s respective qualities when choosing to implement either PROM.

## Introduction

Patient-reported outcome measures (PROMs) quantify various domains of health-related quality of life (HRQOL) from the patient perspective. Patient-reported outcome measures are central in evaluating results of plastic and reconstructive surgery since many interventions focus on improving patient self-perception and quality of life (QOL; Semb et al., 2005; Wormald & Rodrigues, 2018; Dobbs et al., 2019; Geoghegan et al., 2019). High HRQOL, including patient satisfaction with appearance, is one of the most important goals in the treatment of cleft lip and/or palate (CL/P), as clearly expressed by Klassen et al. (2012). Psychological adjustment to CL/P including domains such as developmental trajectory, behavior, emotional well-being, and social experiences are also important components of HRQOL as reviewed by Stock and Feragen (2016).

Implementing PROMs into standard clinical practice is a step closer to the paradigm and ethical approach of patient centered care which is endorsed nationally (Swedish Association of Local Authorities and Regions, 2015) and internationally (Black, 2013; World Health Organization, 2019). The Cleft Hearing Appearance and Speech Questionnaire (CHASQ) developed by the Cleft Psychology Clinical Excellence Network (CEN) and the CLEFT-Q (Klassen et al., 2018b) are 2 CL/P specific PROMs which are used in various countries. Comparison of the results from the 2 different questionnaires and patient opinion about them have not yet been investigated. Considering patient opinion on which PROM allows patients to best express themselves about important outcomes in cleft care is in line with the paradigm shift toward patient-centered care as mentioned earlier.

## Methodological Issues With PROMs in CL/P Related Health Care and Research

One obstacle associated with PROMs in research regarding CL/P is the lack of a widely accepted measurement instrument (Stock et al., 2018; Geoghegan et al., 2019). According to reviews on available PROMs for patients with CL/P by Eckstein et al. (2011) and Klassen et al. (2012), some have been developed ad hoc for a specific study and have not met guidelines for development. Also, most of the reviewed PROMs were not developed specifically for patients with CL/P, as few condition-specific PROMs were available at the time. The reviewed PROMs focused on different domains of HRQOL, for example, speech, facial appearance, or psychological health. Therefore, they would have to be used in combination to represent a holistic assessment of HRQOL in patients with CL/P. A list of proposed PROMs for research and clinical work regarding psychological adjustment, as well as satisfaction with appearance, can be found in a publication by Stock et al. (2020). This work was published for the Global Task Force for Holistic Outcomes, an initiative of the American Cleft Palate-Craniofacial Association.

Methodological issues related specifically to psychological adjustment are discussed by Stock et al. (2018). Challenges listed, which could apply not only to psychological adjustment but to research in all disciplinary fields of CL/P care, are small sample sizes, unclear inclusion/exclusion criteria, lack of consistency in use of PROMs, qualitative research, control and comparison groups, longer-term outcomes research, and interdisciplinary research (Stock et al., 2018). Further limitation in the use of PROMs is the risk of exclusion of participants with low literacy (Wormald & Rodrigues, 2018) as well as the added administrative burden of data collection (Geoghegan et al., 2019).

The rationale for including CHASQ and CLEFT-Q in this study is that both questionnaires are CL/P specific. Both have been studied in various countries (Klassen et al., 2018b; Stiernman et al., 2019a; Kelly, 2020) and both have shown overall acceptable psychometric characteristics according to the unpublished CHASQ User’s Guide by Cleft Psychology CEN from 2015 and Wong Riff et al. (2017). In addition, linguistically validated translations of the questionnaires were available in Swedish. The 2 PROMs also represent 2 different types of questionnaires—CHASQ is briefer, was developed with classical test theory, and produces ordinal-level data. CLEFT-Q is longer, more detailed, was developed with Rasch measurement theory, and produces interval-level data.

## Earlier Comparison of PROMs in Patients With CL/P

Both generic and condition-specific questionnaires have previously been used to explore patient-reported outcomes in populations with CL/P. A drawback with generic questionnaires is that they might not include relevant questions important to individuals with CL/P (Cano & Hobart, 2011; Ricketts et al., 2016; Wormald & Rodrigues, 2018). A condition-specific questionnaire is made specifically with the target population in mind. It has the drawback that the results are hard to compare with norm values of individuals born without CL/P (Cano & Hobart, 2011; Queiroz Herkrath et al., 2015; Wormald & Rodrigues, 2018). For a better understanding of the research field, it is likely that studies of both condition-specific and generic PROMs are necessary (Fitzpatrick et al., 1999; Cano & Hobart, 2011; Crerand et al., 2017).

Comparisons have previously been made between generic, speech- or appearance-specific, and cleft-specific PROMs, see Table 1. One earlier study on HRQOL in patients with CL/P compared a Visual Analog Scale (VAS) and the generic QOL instrument, Pediatric Quality of Life Inventory (PedsQL; Wehby et al., 2014). This study showed that scores from the 2 rating methods correlated well. One strength of the general HRQOL VAS was it’s simplicity, making the method fit for screening patients for low HRQOL. The PedsQL was, on the other hand, more suited for evaluating specific domains of HRQOL. The PedsQL has also been compared with an oral HRQOL questionnaire for children, the Child Oral Health Impact Profile (Broder et al., 2014). Scores from matching domains correlated well in this study. Child Oral Health Impact Profile, being specific to oral health, was found to have greater sensitivity to the need of cleft-related treatment. Pediatric Quality of Life Inventory has also been compared to the velopharyngeal insufficiency (VPI)-specific QOL measure, VPI Effects on Life Outcomes (VELO; Skirko et al., 2012). This study showed correlation between total score, however not all corresponding domains included in respective PROMs correlated. In a further study on VELO, association was also shown with the voice-specific measures Pediatric Voice Outcomes Survey and the Pediatric Voice Related QOL. Visual Analog Scale instruments on speech, swallowing and situation and social interactions were also associated with VELO (Skirko et al., 2013). The study population consisted of 60% patients with cleft palate with or without cleft lip. Velopharyngeal insufficiency Effects on Life Outcomes had higher responsiveness and effect size than the compared PROMs, which may suggest that it is more suitable to detect small changes in VPI-specific QOL after treatment. Results from the Patient-Reported Outcomes Measurement Information Systems (PROMIS) and PedsQL also correlated moderately in patients with cleft lip with or without cleft palate; PROMIS, however, was briefer and showed better readability (Ranganathan et al., 2016).

**Table 1.** Earlier Comparison of PROMs in Patients With CL/P.

Author	Year	n	Age	Main finding
Skirko et al.	2012	58	5-17 years	Scores from total scores of PedsQL and VELO correlated. All corresponding domains did not correlate in patient scores however.
Skirko et al.	2013	84	3-22 years	PVOS, PVRQOL and VAS instrument total scores correlated with VELO. Most corresponding domains also correlated. VELO had higher responsiveness and effect size than the other PROMs.
Wehby et al.	2014	307	5-10 years	Scores from a VAS on general HRQOL and PedsQL correlated well. The VAS was fit for screening patients, PedsQL was more suited for evaluating specific domains of HRQOL.
Broder et al.	2014	1200	7-18 years	Scores from matching domains of PedsQL and COHIP correlated. COHIP had greater sensitivity to the need of cleft related treatment.
Ranganathan et al.	2016	93	5-20 years	Scores from PROMIS and PedsQL correlated moderately. PROMIS was briefer and showed better readability.

Abbreviations: CL/P, cleft lip and/or palate; COHIP, Child Oral Health Impact Profile; HRQOL, health-related quality of life; PedsQL, The Pediatric Quality of Life Inventory; PROMIS, Patient Reported Outcomes Measurement Information Systems; PROMs, patient-reported outcome measures; PVOS, Pediatric Voice Outcomes Survey; PVRQOL, Pediatric Voice Related Quality of Life; VAS, Visual Analog Scale; VELO, Velopharyngeal insufficiency Effects on Life Outcomes.

In summary, total scores and the majority of corresponding domains within different PROMs have correlated well within the CL/P population irrespective if the PROM was generic, condition specific, very detailed, or a simple VAS scale (Skirko et al., 2012; Skirko et al., 2013; Broder et al., 2014; Wehby et al., 2014; Ranganathan et al., 2016). Briefer PROMs were shown to be easier to complete and more suitable as screening tools (Ranganathan et al., 2016). Longer, condition specific and more detailed instruments had higher sensitivity in evaluating issues in specific domains of HRQOL and the need for further CL/P-related treatment (Skirko et al., 2013; Broder et al., 2014; Wehby et al., 2014). Association between PROMs on satisfaction with appearance as well as CHASQ and CLEFT-Q have not yet been investigated.

## Clinical Implementation of a PROM

A PROM that is valid for research and at the same time is perceived as a useful tool in clinical work may be used more widely and be easier to implement (Cano et al., 2009). Clinicians could also use the PROM as a tool to initiate conversation (Stiernman et al., 2019a). Further, it could be important as an icebreaker for difficult-to-discuss topics in a health care setting (Rotenstein et al., 2017). Health care professionals may find this element useful, since they have expressed challenges in discussing psychosocial health and satisfaction with patients with CL/P (Stiernman et al., 2019b). Patients may also more readily complete PROMs if they perceive the questionnaire poses questions that are important to them and their treatment (Cano et al., 2009). Other factors that could affect whether patients complete an entire PROM is the degree of psychological discomfort caused by the PROM and the amount of time and effort required (Fitzpatrick et al., 1999; Aaronson et al., 2002).

The aim of the study was:

1. To investigate the correlation of corresponding scores between 2 existing CL/P specific PROMs, CHASQ and CLEFT-Q.
2. To investigate patient opinions on CHASQ and CLEFT-Q.

Ethical approval was obtained from the Ethical Board in Lund, Sweden (Reference nr: 2015/799 and 2015/800).

## Methods

### Participants

Treatment of patients born with CL/P in the southern part of Sweden is offered by the CL/P team in Skåne University Hospital where the study was conducted. Patients were recruited on routine visits to the center. Patients who underwent treatment at the CL/P center during the recruitment period of the study were also recruited. Informed consent was obtained from all participants. Participants were given information about the study verbally and in written form. For participants younger than 15 years, consent was obtained from the parents. Questionnaires were not anonymized.

The first author, or the social counsellor at the clinic, handed out 80 sets of CHASQ and CLEFT-Q in paper form to patients. Of these, 19 sets (24%) were returned. In addition, 14 sets of questionnaires were collected as part of other studies (Klassen et al., 2018b; Stiernman et al., 2019a) and were included in the study data. Recruitment process and inclusion criteria were the same for these additional 14 participants. Parents were informed that they could help their children to fill out the questionnaires. It was stressed that the child’s opinion should be represented by the answers. In total, 33 participants with CL/P answered both questionnaires CHASQ and CLEFT-Q, within the same day or within a couple of days. The age of the children and young people ranged from 10 to 19 years, mean age was 13 years. Thirteen (39%) females and 20 (61%) males participated. The population of 33 participants constitutes a convenience sample.

### Inclusion Criteria

Inclusion criteria were ages between 10 and 19 years and the ability to read and write Swedish. All types of CL/P were included. Distribution of diagnoses is listed in Table 2. Results for CHASQ have been calculated according to the criteria specified in the CHASQ User’s Guide from 2015. Patients with all types of CL/P answer all 15 items. For example, both patients with and without a visible cleft answer all items, including those focused on appearance. Results for CLEFT-Q have been calculated according to instructions on the questionnaire. For example, patients with cleft palate only (CP) do not answer the scale *Cleft lip scar*, patients with no cleft palate do not answer the scales *Speech function* and *Speech distress*, and patients younger than 12 years do not answer the scale *Jaws.* Data in this study have been analyzed in the same way to facilitate comparison with these earlier established norms. Additional calculations were performed on the 26 patients with visible clefts in this study to examine results specifically regarding appearance. In the subpopulation of children and young people with visible clefts, age ranged from 10 to 19 years, mean age was 13 years, 8 (31%) were females and 18 (69%) were males. The total comparison adjusted score was calculated to be able to compare results with a Swedish noncleft control population comprising 56 participants aged 9 to 20 years (Stiernman et al., 2020). The total comparison adjusted score excludes item nr 15 “Overall how noticeable do you feel your cleft is to other people?” from the total score since noncleft participants cannot answer this item.

**Table 2.** Diagnoses Represented in the Study Population.

Diagnose	n
Cleft palate only	7 (21%)
Cleft lip only	9 (27%)
Cleft lip and alveolus	3 (9%)
Unilateral cleft lip and palate	7 (21%)
Bilateral cleft lip and palate	7 (21%)
Total	33 (100%)

### Cleft Hearing Appearance and Speech Questionnaire

Cleft Hearing Appearance and Speech Questionnaire is a modified version of the Satisfaction with Appearance questionnaire (SWA). Satisfaction with Appearance questionnaire was designed by the Cleft Psychology Special Interest Group of the Craniofacial Society of Great Britain and Ireland specifically for patients with facial disfigurement (Emerson et al., 2004). Special Interest Group was changed into Cleft Psychology CEN and SWA was thereafter modified to CHASQ. It consists of 15 items regarding satisfaction with different features of the face, hearing, and speaking. Score for each item ranges from 0 to 10 points. Higher scores indicate higher satisfaction. Norm values of CHASQ are based on a population of 867 patients, between the ages of 10 and 20 years with CL/P in the United Kingdom. These are presented in the User’s Guide from 2015. For the total score of items in CHASQ, the median was approximately 124 points.

### CLEFT-Q

CLEFT-Q was developed as a CL/P specific questionnaire through a process of review of existing PROMs in the field (Klassen et al., 2012), by qualitative research of patients with CL/P from different countries (Tsangaris et al., 2017; Wong Riff et al., 2018), field testing in 12 countries and, finally, analysis with Rasch measurement theory (Klassen et al., 2018b). It consists of 12 scales and 1 checklist with 6 to 12 items each. The answers from each scale must be converted using a conversion table to obtain the final score. Scores range from 0 to 100 points. Higher scores indicate higher satisfaction. Norm values of the CLEFT-Q in different age-groups and CL/P diagnosis have been established based on 2434 individuals aged 8 to 29 years from 12 different countries (Klassen et al., 2018b). Norm values of the scales for different age-group and CL/P diagnosis were stated as the median score per sample.

### Survey on Patient Opinion on CHASQ and CLEFT-Q

An extra survey included in the study contained closed- and open-ended questions comparing the 2 instruments. This exploratory part of the study specifically asked for patient opinion about which questionnaire they liked best, which was easier to answer, and which questionnaire they believed would best inform health care professionals.

### Statistics

Nonparametric tests were used due to the fact that scores produced by CHASQ are ordinal-level data. This means that intervals on different parts of the scale do not necessarily imply equal change in satisfaction with hearing, appearance, or speech (Cano & Hobart, 2011). Spearman correlation was used to test correlations. Differences in CHASQ and CLEFT-Q scores and age between subgroups of participants were calculated with Mann-Whitney *U* test. For all statistical analyses, *P* <.05 (2-tailed) was considered to indicate a significant difference. All analyses were calculated with IBM SPSS Statistics 26.

## Results

### Correlation Between Corresponding Items and Scales in CHASQ and CLEFT-Q

Mean CHASQ items, total, comparison adjusted total scores, and CLEFT-Q scale scores are presented in Tables 3 and 4. Mean CHASQ item scores ranged from 7.7 to 9.5 out of 10 points. Mean CLEFT-Q scale scores ranged from 66 to 87 out of 100 points. All items on satisfaction with appearance of the face and specific parts of the face in CHASQ correlated moderately to very strongly with corresponding scales in CLEFT-Q (Table 5). Additional correlations between corresponding items and scales on appearance were performed exclusively with the 26 patients included in the study with visible clefts (excluding CP). These results showed moderate to very strong correlations and were similar when patients with CP were not excluded from the analysis (Table 6). Speech item in CHASQ did not correlate significantly with *Speech function scale* or *Speech distress scale* in CLEFT-Q (Spearman ρ 0.276, *P* = .226).

**Table 3.** Minimum, Maximum, Mean Scores, and Standard Deviation for CHASQ Items, Total and Comparison Adjusted Total Score.

CHASQ item	n	Minimum	Maximum	Mean	Std deviation
1. Face	33	4	10	8.2	1.9
2. Whole appearance	33	2	10	8.3	2.1
3 Side view/profile	33	0	10	8.1	2.4
4. Good looking	33	3	10	8.1	1.8
5. Nose	33	0	10	7.7	2.4
6. Lips	33	0	10	8.0	2.5
7. Chin	33	5	10	9.4	1.2
8. Teeth	33	3	10	7.9	2.1
9. Cheeks	33	6	10	9.4	1.1
10. Hair	33	6	10	9.5	1.0
11. Ears	33	4	10	9.2	1.4
12. Eyes	33	7	10	9.5	0.8
13. Speech	33	0	10	8.4	2.0
14. Hearing	33	3	10	8.7	2.0
15. How noticeable to others	33	4	10	7.9	2.0
Total items score	33	84	150	128	17
Comparison adjusted total score	33	76	140	120	17

Abbreviations: CHASQ, Cleft Hearing Appearance and Speech Questionnaire; Std, standard.

**Table 4.** Minimum, Maximum, Mean Scores, and Standard Deviation for CLEFT-Q Scales.

CLEFT-Q scale	n	Minimum	Maximum	Mean	Std deviation
Face	33	42	100	73	18
Nose	33	36	100	72	21
Nostrils	32	0	100	71	29
Teeth	33	31	100	66	23
Jaws	19	17	100	87	22
Lips	32	15	100	77	22
Cleft lip scar	26	30	100	78	23
Psychological function	26	26	100	79	18
School function	30	33	100	81	18
Social function	33	45	100	79	17
Speech distress	21	36	100	83	20
Speech function	21	8	100	78	23
Eating and drinking	33	0 problems	6 problems	-	-

Abbreviation: Std, standard.

**Table 5.** Correlation Between CHASQ Items and Corresponding CLEFT-Q Scales Calculated With Spearman Correlation.

CHASQ item–CLEFT-Q scale	n	Spearman ρ	*P*
CHASQ Face item–CLEFT-Q Face scale	33	0.816	.001
CHASQ Nose item–CLEFT-Q Nose scale	33	0.712	.001
CHASQ Nose item–CLEFT-Q Nostrils scale	32	0.562	.001
CHASQ Lips item–CLEFT-Q Lips scale	32	0.646	.001
CHASQ Lips item–CLEFT-Q Lip scar scale	26	0.507	.008
CHASQ Teeth item–CLEFT-Q Teeth scale	33	0.735	.001
CHASQ Speech item–CLEFT-Q Speech function scale	21	0.276	.226
CHASQ Speech item–CLEFT-Q Speech distress scale	21	0.294	.196

Abbreviation: CHASQ, Cleft Hearing Appearance and Speech Questionnaire.

**Table 6.** Correlation Between CHASQ Items and Corresponding CLEFT-Q Scales Calculated With Spearman Correlation Including Only Patients With a Visible Cleft in Calculations Regarding Appearance.

CHASQ item–CLEFT-Q scale	n	Spearman ρ	*P*
CHASQ Face item–CLEFT-Q Face scale	26	0.799	.001
CHASQ Nose item–CLEFT-Q Nose scale	26	0.677	.001
CHASQ Nose item–CLEFT-Q Nostrils scale	25	0.553	.006
CHASQ Lips item–CLEFT-Q Lips scale	25	0.661	.001
CHASQ Lips item–CLEFT-Q Lip scar scale	26	0.507	.008
CHASQ Teeth item–CLEFT-Q Teeth scale	26	0.756	.001

Abbreviation: CHASQ, Cleft Hearing Appearance and Speech Questionnaire.

### Patient Opinion on CHASQ and CLEFT-Q

A slight majority, 15 participants (58%, n = 26), liked CLEFT-Q more than CHASQ. Eighteen participants (69%, n = 26) thought CHASQ was easier to complete. However, 19 (76%, n = 25) thought that CLEFT-Q would better inform health care professionals. Patient opinion on the 2 questionnaires with closed-ended items is summarized in Table 7.

**Table 7.** Answers to Closed-Ended Questions in Survey Regarding Patient Comparison of the CHASQ and the CLEFT-Q.

Closed ended-questions	n	Patients preferring CLEFT-Q	Patients preferring CHASQ	Patients who answered “No difference”
Which questionnaire did you like the most?	26	15 (58%)	9 (35%)	2 (8%)
Which questionnaire did you think was easiest to complete?	26	7 (27%)	18 (69%)	1 (4%)
Both the questionnaires are meant to help health care professionals understand what you think about yourself and your cleft. Which questionnaire do you think works best?	25	19 (76%)	4 (16%)	2 (8%)

Abbreviation: CHASQ, Cleft Hearing Appearance and Speech Questionnaire.

Participants could embellish their answers with written responses to the open-ended items in this survey. They gave various reasons as to why they preferred either instrument. A common theme regarding CLEFT-Q was that they appreciated the depth and detail offered by the more elaborate scales. Scales regarding feelings and social health were additionally mentioned as important. Patients thought that the instrument would provide more detailed and broader information to health care professionals.

*CLEFT-Q was more thorough on every part of the face, and feelings in general. If health care professionals really are interested in my opinions then CLEFT-Q probably provides better insight, even though CHASQ provides a better overview. (19-year-old woman)*

Participants who liked CHASQ seemed to prioritize ease of completion over breadth or detail. Some parents preferred CHASQ because their child had very little problems with the cleft or had only a cleft of the soft palate; CLEFT-Q therefore had many questions that did not feel relevant to them. It is suspected that parents of the younger participants wrote some responses since the child was referred to in third person. These comments revealed a possible concern that too many items about feelings and appearance would upset their child.

*Fewer questions. Only questions on two pages. The questions were easier to answer. (10-year-old boy)*

*The questions that are general in CLEFT-Q regard parts of the face which are normal if you only have had an isolated cleft palate and create a mess in a child’s head since they can find faults which are not there. (Parent of a 10-year-old boy)*

## Discussion

### Correlation Between Corresponding Items and Scales in CHASQ and CLEFT-Q

Results from corresponding items on satisfaction with appearance in CHASQ and scales in CLEFT-Q correlated moderately to very strongly. Correlations between corresponding domains in the CL/P population have been shown for other domains of HRQOL (Broder et al., 2014; Wehby et al., 2014; Ranganathan et al., 2016). Scores from the speech item in CHASQ did not correlate significantly with scores from the *Speech function* scale or the *Speech distress* scale in CLEFT-Q. This finding is in contrast with 2 earlier studies on HRQOL in patients with VPI (Skirko et al., 2012; Skirko et al., 2013). A possible reason for this finding could be the limited population. The number of participants in this part of the analysis was 21, compared with the number of participants in the above-mentioned studies (n = 58 and n = 84, respectively; Skirko et al., 2012; Skirko et al., 2013). The low number of participants was a result of the exclusion of patients without a cleft palate.

The total combined score of 128 of CHASQ in this study was similar to earlier established total combined score of approximately 124 points in a British norm population with CL/P in the CHASQ User’s Guide from 2015. On a single item level, the mean British norm for item nr 1 “How happy are you with how your face looks” was approximately 8.3 points, which is similar to the mean score in this study of 8.2 points for item nr 1. The British norm for item nr 13 “How happy are you with your speech?” was approximately 8.7, which corresponds to the mean score of 8.7 in this study. The scores were also similar to a Swedish subpopulation of 47 participants, aged 7 to 19 years, score of 124 points in a European pilot study on CHASQ (Stiernman et al., 2019a). On a single item level, the mean Swedish pilot study score for item nr 1 was 7.9 points and the mean score for item nr 13 was 8.5. Kelly and Shearer (2020) presented the mean single item score of item nr 1 and nr 13 from CHASQ in a British population aged 10 to 15 years. Their scores were approximately 8.1 points for item nr 1. An exception was the subpopulation of 10-year-old children with CP who scored 9.26 points and the subpopulation of 15-year-old adolescents with CLP who scored 6.25 points. Their score of item nr 13 was approximately 8.8 points for item nr 13 for 10-year-olds, and for 15-year-olds, it was 8 points. In summary, total and single item scores on CHASQ in this study were similar to earlier norm values and earlier studies (Stiernman et al., 2019a; Kelly & Shearer, 2020).

Comparison adjusted total CHASQ scores have earlier been presented for a Swedish non-CL/P control population by Stiernman et al. (2020). Their comparison adjusted total CHASQ score for the non-CL/P control population was 119 points. These results were similar to the comparison adjusted total CHASQ score in this study of 120 points.

The scores in this study on each scale in CLEFT-Q were similar to norm values, except for the scale Cleft lip scar, where patients in this study scored approximately 10 points higher (Klassen et al., 2018b). The reason for the difference in score on the *Cleft lip scar* scale is unclear but could be due to the small sample size.

### Patient Opinion on CHASQ and CLEFT-Q

Both instruments have shown strengths and limitations. A majority of participants thought that CHASQ was easier to complete. From the perspective of everyday clinic usage, CHASQ has also been found to be an easy to use and a brief instrument (Stiernman et al., 2019a). CHASQ can therefore be assumed to be easier to implement than CLEFT-Q. Most participants, however, seemed to think that CLEFT-Q was a better way to inform health care professionals and may therefore be completed by patients to a greater extent due to high face validity. This may partly be explained by the fact that the scales and items in CLEFT-Q were generated with patient input through interviews (Tsangaris et al., 2017; Wong Riff et al., 2018). The themes represented by the scales were clearly appreciated and regarded as important. In clinical audit and research, it is essential to measure aspects of health and care that are important to patients (Wong Riff et al., 2018). CLEFT-Q is also hypothesized to have higher responsiveness since it is more detailed (Wormald & Rodrigues, 2018). Both CHASQ and CLEFT-Q have the potential to inform health care professionals and to initiate important discussion with patients in a clinical setting. Since difficulty in discussing psychosocial topics has been recorded in health care professionals working with patients with CL/P (Stiernman et al., 2019b), implementation of CLEFT-Q may facilitate discussion on precisely these topics.

Clinicians will have to consider the strengths and limitations in relation to both questionnaires as well as the resources of the hospital system when choosing to implement either PROM. Collaboration between clinicians and researchers has earlier been suggested for implementation of PROMs with both “face validity and scientific rigor” (Stock et al., 2020). Both PROMs have the potential to allow CL/P teams to enhance patient–clinician communication (Wormald & Rodrigues, 2018) and shared decision-making between patients and clinicians (Dobbs et al., 2019).

### Limitations

The low rate of participation (approximately 24% of the questionnaires were returned) posed a risk for inclusion bias. There were also patients who did not complete all the items in CHASQ or the survey on patient opinion on CHASQ and CLEFT-Q, which further decreased the sample size. CLEFT-Q instructs patients to fill out certain scales, depending on cleft type and age. This decreased the sample size for certain parts of the analysis. There is also a risk that some patients thought the PROMs were psychologically challenging (due to dissatisfaction with appearance or speech) and therefore did not fill out the questionnaires. Thus, these individuals may have been underrepresented in this study. No follow-up was undertaken to remind the participants to return the questionnaire. This could, in part, contribute to the low participation rate. The age range, cleft types, and sex further decreased the sample size in these respective groups. An earlier study found poorer outcomes on CLEFT-Q in older participants, participants with a visible cleft, as well as in females (Klassen et al., 2018b). Further research on this subject with a larger population would be valuable to allow for stratification for these subpopulations. Future studies could also include information on background variables such as socioeconomic status or level of education to ensure representativeness of the results.

CLEFT-Q does not include a scale on hearing, or how noticeable the patient thinks his/her cleft is. Comparison between questionnaires on these items was therefore not possible. Unlike CLEFT-Q, CHASQ does not have items regarding school and social life, psychology, eating, or drinking, and therefore comparison between questionnaires on these scales was also not possible. For a broader holistic assessment of patient-reported outcomes, both CLEFT-Q and CHASQ could be combined with other PROMs as suggested by International Consortium for Health Outcomes Management (ICHOM; Allori et al., 2016) and the Global Task Force for Holistic Outcomes (Stock et al., 2020).

It was clear from the written responses that the child had not answered the open-end items. It is more likely that a parent of the child wrote the comments on the survey. The closed-ended items on comparison of CHASQ and CLEFT-Q, however, are still believed to reflect participant opinion. This cannot be certain however, since it was not possible to monitor the completion of the PROMs. The order of completion of the 2 PROMs was also not controlled. The order of completion might have affected the rate of noncompleted items in CHASQ. Scores in CLEFT-Q could however be calculated if the missing data did not exceed 50% of a scale’s items (CLEFT-Q Users’ Guide, 2018).

### Future Considerations

CLEFT-Q was handed out to all participants in its entire form. However, only patients with a cleft lip, alveolus, and palate between 12 and 18 years of age completed all the scales. The instructions, for example, for the scale regarding patient feelings about the lip scar, explain that the scale should only be completed if the patient has a cleft lip. Further, in recommendations from the ICHOM for CL/P patients, 8-year-olds should only complete 4 CLEFT-Q scales, 12 year olds should complete 7 scales, and 22 year olds (or when the patient ends treatment at the CL/P center) should complete 6 scales (Allori et al., 2016). This recommendation reduces the burden of completion for the patient and could thus possibly influence their opinion on the ease of completion. If the instrument had been administered electronically, scales could have been automatically presented to patients depending on age and diagnosis. Computerized adaptive testing (CAT) has been evaluated with CLEFT-Q (Harrison et al., 2019). The study showed that 97% accuracy of final result could be obtained by using only 43 items (39.2%) of the 110 items in the full scale. Utilizing CAT in administration of CLEFT-Q could further reduce burden on patients.

During the international field test of CLEFT-Q, patients from 12 countries reported how the instrument had impacted them (Klassen et al., 2018a). Of 2047 patients, 88% responded that they liked answering the questionnaire and 12% responded that they did not like answering it. Of 1922 patients, 25% responded that the questions made them feel better about the way they looked, 72% felt the same way, and 3% felt worse after answering the questionnaire. Finally, of 2048 patients, 77% did not feel upset or unhappy about the way they looked, 20% felt a little upset or unhappy, and 3% felt very upset or unhappy after answering the questionnaire. In conclusion, the majority of patients were happy to fill out the scales in CLEFT-Q and only a few were upset by it.

The occurrence of distressed participants is a concerning issue since the principle of minimization of malevolence states that health care providers and medical researchers should not harm their patients. This issue should, however, be weighed against the fact that the majority of patients liked to complete the instrument as well as the potential benefit for all patients to be able to report their opinion on surgical outcome and psychosocial health (World Medical Association, 2013). Patients who are most likely to be distressed when completing the instrument may also be more likely to be those who would not spontaneously speak about their feelings in a clinical setting. These patients could possibly be those in most need of further psychological support. Patients should be able to answer the PROM voluntarily. An important aspect to consider is that a clear pathway of referral should be available to patients if a PROM reveals that psychological treatment or counseling is required.

## Conclusion

Corresponding scores regarding appearance gathered with CHASQ and CLEFT-Q correlated moderately to very strongly. Corresponding scores on speech did not correlate significantly. A majority of participants thought that CHASQ was easier to complete than CLEFT-Q. A slight majority of participants liked CLEFT-Q over CHASQ and thought that it better informed health care professionals. Clinicians will have to consider the respective qualities of each questionnaire when choosing to implement either PROM.
